# Gender differences of depression and anxiety among social media users during the COVID-19 outbreak in China:a cross-sectional study

**DOI:** 10.1186/s12889-020-09738-7

**Published:** 2020-11-04

**Authors:** Fengsu Hou, Fengying Bi, Rong Jiao, Dan Luo, Kangxing Song

**Affiliations:** 1 Department of Public Health, Shenzhen Kangning Hospital, No.1080 Cuizhu Road, Luohu District, Shenzhen, 518020 Guangdong China; 2 Department of Social Medicine, Xiangya School of Public Health, Central South University, No.110 Xiangya Road, Kaifu District, Changsha, 410078 Hunan China; 3 The First Clinical College, Hainan Medical University, No.3 Xueyuan Road, Longhua District, Haikou, 570100 Hainan China; 4 Department of Cardiology, The First Medical Center of Chinese PLA General Hospital, No.51, Fucheng Road, Haidian District, Beijing, 100853 China

**Keywords:** COVID-19, Depression, Anxiety, Gender differences, Social media use

## Abstract

**Background:**

Studies have shown that the outbreak of infectious diseases would result in mental health problems. Females are in greater risk for psychological problems than males. The present study investigated gender differences of depression and anxiety and explored associated factors during the COVID-19 epidemic among Chinese social media users.

**Methods:**

We recruited 3088 participants through social media cross China. Participants completed sociodemographic and the COVID-19 epidemic related questions, the 2-item Patient Health Questionnaire (PHQ-2), and the 2-item Generalized Anxiety Disorder Scale (GAD-2), the Chinese version of the 10-item Connor-Davidson Resilience Scale. We applied Chi-square test and ANOVA for data description and linear regression analysis for exploring factors associated with depression and anxiety.

**Results:**

Of 3063 participants eligible for analysis, the total prevalence of depression and anxiety was 14.14 and 13.25%. Females were experiencing more severe stress and anxiety symptoms, while males showed better resilience to stress. The severity of depression symptoms would decrease with the increase of age resilience, and it would increase if being unemployed, feeling less adapted, being more stressed. The severity of anxiety symptoms would decrease with higher education and better resilience, and it would increase if being female, spending over 60 min on COVID-19 related information, less adapted, and being more stressed.

**Conclusion:**

The findings show the increased prevalence of depression and anxiety in Chinese population during the COVID-19 epidemic, and females are experiencing more severe anxiety symptoms than males. As social media is the current main resource of information related to COVID-19, interventions should be implemented to help users to limit the time they spend on social media and to get key information related to the epidemic from authoritative and authentic resource to avoid infodemic and prevent mental health problems.

## Background

The outbreak of 2019 novel coronavirus disease (COVID-19), which first emerged in Wuhan, Hubei Province, China, then quickly spread to the rest of China and other countries, has been recognized by the World Health Organization (WHO) as a global public health emergency [1, 2]. Since January 25th, 2020, the authorities of provinces, municipalities and autonomous regions in China have initiated first-level responses to major public health emergent events, including strict public transport control, forbidden to any kind of gathering, closing public places, mandatory mask wearing and requiring self-quarantine for all. During the physical social isolation, it was estimated that the Chinese spent increased time on social media such as TikTok, Weibo and WeChat which was estimated to be7.6 h per day (53.2 h per week), in comparison, it was 27.9 h per week in 2019 [3, 4]. Further, the convenience and the speed of disseminating information on the social media result in overwhelming news including the prevalence, mortality, confirmed cases and high contagion of COVID-19 as well as tragic report of ceases patients and families. Studies report the stress stemming from quarantine and overwhelming news associates with depression and anxiety [5–8]; however, little is known about since the outbreak.

It has been well established that women are in greater risk for psychological problems than men, because of the interactions between biological factors and social determinants including gender stereotypes and roles, social stigma and inequity, and social autonomy [9–11]. The latest national study in China reported the 12-month prevalence of any mood disorders and any anxiety disorder was 3.50 and 4.80% in males, 4.60 and 5.20% in females [12]. The gender differences lead to a variety of physical and mental health outcomes. For example, depressive disorder is the 16th leading cause of burden of disease for male in China and it is the 5th for female; in comparison, anxiety disorder is the 17th leading cause of burden of disease for females in China and it is not in the top 25 leading cause of burden of disease for males [13]. More importantly, the comorbid depression and anxiety is more common in females [14, 15]. Females with mental disorders are in increased risk for experience intimate partner violence (IPV) that is not well studied and understood in China due to the cultural doctrines of “face” (personal dignity, prestige, and self-perception) and “jia chou bu ke wai yang” (one should not reveal family disgrace to outsiders), which would result in depression and anxiety in return [16–22]. In specific, given attaining or maintaining face is related to reactions toward conflicts, males may feel losing face if their partners were experiencing mental disorders due to the public stigmatization, and violence against women has then become a possible way to save and gain face to show off the dominance and masculine [18, 19, 23].

During the epidemic, it is inevitable to ignore the gender differences in users behaviors of social media and its potential impact on psychological well-being [24–27]. For example, males use blogs, media-sharing sites, social questioning and answering and user reviews more frequently than females [28]; however, little is known about the gender differences in seeking COVID-19 information and its impact on psychological stressing state.

In this study, we aim to investigate the gender differences of depression and anxious symptoms, and to explore factors associated with mental health during the COVID-19 epidemic among Chinese social media users.

## Method

### Sample and sampling

Since February 20th, 2020, the study team started to recruit participants by a snowball like convenient sampling method. First, research team members sent out recruitment advertisement, which contained a welcome note and a link to the on-line questionnaire, on two monopolized and universal social instant communication platforms in China, WeChat and QQ run by Tencent [29, 30]. The first wave participants from diverse geographical locations of origin, were directly from research team members’ social network based on Moment (similar to Facebook Timeline or Twitter News Feed service and a bonded service to Wechat) and Q-Zone (similar to MSN Space or MySpace and a bonded service to QQ), and the research team was based in Hunan Province adjacent to Hubei Province. Once finished the survey, participants were encouraged to disseminate the advertisement through their social network on Moment or Q-Zone to recruit the next wave of participants who would be the 2nd, 3rd and even 4th degree contacts of the first wave participants. The recruitment procedure continued till the team terminated on February 27th, 2020.

Of note, WeChat and QQ share similar featured functions including texting, voice and video calling, group chat, photo sharing, games, map navigation, ticket booking, financial managing, payment and so forth, especially, WeChat is described as the app for everything indicating the apps are developed for anybody to use at anytime and anywhere for anything; due to the features, WeChat and QQ have reached about 11 and 7 billion active users in the first quarter of 2020 [31–33].

During recruitment, we did not set inclusion criteria for potential participants. However, we excluded participants during data preparation if they were under 18 years old or living outside the mainland of China. Meanwhile, all participants must read and agree the informed consent before the survey. In total, we have received 3088 copies of on-line survey.

The protocol including the on-line consent was reviewed and approved by the Ethics Committee of Xiangya School of Public Health of Central South University.

### Measures

We developed the sociodemographic questionnaire to collect participants’ characteristics including age, gender, education level, marriage status, occupation and health status. We also developed a questionnaire to collect information that may be related to the COVID-19 epidemic and mental health, including the province of residence, quarantine status, number of people living together, time spent on seeking for COVID-19 related information, main source of COVID-19 related information and adaption to the quarantine.

We applied the 2-item Patient Health Questionnaire (PHQ-2) to investigate depressive symptoms. The PHQ-2, extracted items from the 9-item Patient Health Questionnaire (PHQ-9), is a 4-point scale ranging from 0 (not at all) to 3 (nearly every day) [34]. It evaluates the frequency of “feeling down, depressed or hopeless” and “little interest or pleasure in doing things” over the past 2 weeks. The total score ranges from zero to six, and a score greater than three indicates depression. The PHQ-2 has been validated and used in China [35, 36]. The Cronbach’s alpha was 0.774 in this study.

We applied the 2-item Generalized Anxiety Disorder Scale (GAD-2) to investigate anxiety symptoms. The GAD-2, extracted items from the 7-item Generalized Anxiety Disorder Scale (GAD-7), is a 4-point scale ranging from 0 (not at all) to 3 (nearly every day) [37]. It evaluates the frequency of “feeling nervous, anxious, or on edge” and “not being able to stop or control worrying” over the past 2 weeks. The total score ranges from zero to six, and a score greater than three indicates anxiety. The GAD-2 has been validated and used in China [38, 39]. The Cronbach’s alpha was 0.800 in this study.

We applied the Chinese version of the 10-item Connor-Davidson Resilience Scale (CD-RISC-10) to investigate participants’ resilience to stress. The CD-RISC-10, extracted items from the Chinese version of 25-item Connor-Davidson Resilience Scale, is 5-point scale ranging from 0 (not true at all) to 4 (true nearly all the time), and it has been validated in Chinese sample [40]. It evaluates abilities to adapt to change, to cope with stress, to stay focused and think clearly, to deal with things and matters, failures, and unpleasant feelings. The total score ranges from zero to forty, and a higher score indicates greater resilience to adverse events. The Cronbach’s alpha was 0.9360 in this study.

At last, we applied one 10-point item to evaluate participants’ perceived stress, and, from one to ten, a higher self-rated score indicates a higher level of stress.

### Data analysis

In this study, we analyzed data with R (version 3.5.1) and set the statistical significance at 0.05 [41].

After recruitment, we transported data from on-line to database for analysis. We have set up a rule in the on-line survey system that all participants must provide answers to all questions to complete, hence there was no missing cases. However, during data cleaning, we excluded 10 participants living outside of China and 15 participants under 18 years old. Eventually, there were 3063 copies of on-line survey eligible for analysis. Of the 3063 participants, there were 1327 males and 1736 females.

For further analysis, we recoded several variables. First, participants’ occupation was categorized into four classes: front-line medical personnel (doctors, nurses and other medical personnel who had ever worked at the front-line against the epidemic), general medical personnel (doctors, nurses and other medical personnel who did not work at the front-line), employed (employees in other professions outside of medical area), unemployed (students and others who did not have any income). Second, based on the province of participants’ residence and its distance to Hubei Province, we recoded the variable as distance to Hubei Province by four categories: Hubei Province, first class areas (provinces sharing boundary with Hubei Province), second class areas (provinces sharing boundary with those in the first class), third class areas (the rest provinces in China and foreign residences). Third, we recoded participants’ health status into two classes: healthy and any diseases (with any kind of diagnosed physical or psychiatric diseases). Forth, the main source of COVID-19 related information was recoded into two categories: traditional media (including TV, radio broadcast, newspaper, magazines, friends and colleagues), social media (including website, Q & A forum and apps).

### Analytic plan

We compared the characteristics between male and female participants by one-way ANOVA or Wilcoxon rank test (if the data were of skewed distribution) for continuous variables, Chi-square test for categorical variables, and Fisher’s exact test if necessary. Descriptive analysis were conducted by R package “psych” [42].

We created dummy variables for categorical variables first, then conducted univariate linear regression analysis between depression/anxiety and potential associated variables first, and then to conduct multivariate linear regression analysis with variables of significance in the univariate analysis. In multivariate analysis, we adopted a stepwise backward strategy. We chose the Adjusted R-squared and the F-value to assess the fitness of models, and higher values indicated better model fitness. Analysis were conducted by R package “car” [43].

## Results

### Overall

In this study, the age of participants ranged from 18 to 91 years old with a mean of 37.73 ± 13.38. There were 2014 (65.75%) participants were married. A majority of participants (1999, 65.26%) had received bachelor’s degree, 453 of them (14.79%) had received master’s degree and above, and 611 of them (19.95%) have finished high school or received less than 9 years education. Among all participants, 216 of them (7.05%) were first line medical personnel in the epidemic, 348 of them (11.36%) were general medical personnel, 2410 of them (76.68%) were employed, and 89 of them (2.91%) were unemployed. Most participants (2416, 78.88%) were healthy and did not have any diagnosed diseases, and 647 of them (21.23%) had one or more diagnosed physical or psychological diseases. There were a small portion of participants (63, 2.06%) residing in Hubei Province, 1084 of them (35.39%) residing in first class areas, 984 of them (32.13%) residing in second class areas and 932 of them (30.43%) residing in third class areas.

Among all participants, 5 of them (0.16%) were under quarantine for treatment of COVID-19, 839 of them (27.39) were under quarantine for observation, and 2219 of them (72.45%) were under home quarantine. Most of participants (85.37%) were living with 2 to 5 people, 225 of them (7.35%) were living alone and 223 of them (7.28%) were living with 6 people and above. Every day, there were 984 participants (32.13%) spending less than 30 min, 1445 participants (47.18%) spending 30 to 60 min, and 634 (20.70%) participants spending more than 60 min on seeking for COVID-19 related information. Only a minority of participants (126, 4.11%) mainly used traditional media as source of information, while the rest (2937, 95.89%) mainly used social media. There were 1746 participants (50.00%) were very adapted to the current working and living condition, 741 participants (24.19%) were adapted, and 576 participants (18.81%) were not adapted.

The score of PHQ-2 ranged from 0 to 6 with a mean of 1.30 ± 1.41. The prevalence of depression was 14.14% (433/3063); and it was 14.92% (198/1327) in males and 13.52% (235/1738) in females, however the difference was not significant. The score of GAD-2 ranged from 0 to 6 with a mean of 1.28 ± 1.44. The prevalence of anxiety was 13.25% (/3063); and it was 21.21% (162/1327) in males and 14.04% (244/1738) in females, however the difference was not significant. The score of CD-RISC-10 ranged from 0 to 40 with a mean of 28.58 ± 8.08. The score of self-rated stress ranged from 0 to 8 with a mean of 3.45 ± 2.39.

Gender differences were observed in several aspects. Among sociodemographic characteristics, we found the marriage rate was higher in males (68.43% vs. 63.64%) and the rate of participants living with 2 to 5 people was higher in females (87.40% vs. 85.59%). The rate of using traditional media as main source of information in males (5.12% vs. 3.34%). The rate of reporting very adapted and adapted was higher in females (58.80 and 24.63%), while it was 54.56 and 23.59% in males. We also found females reported higher score in self-rated stress and GAD-2 than males (3.53 ± 2.39 vs. 3.34 ± 2.37, 1.35 ± 1.44 vs. 1.18 ± 1.43), indicating they were experiencing more severe stress and anxiety symptoms. Meanwhile, males showed better resilience to stress than females, and the mean score of CD-RISC-10 was 29.63 ± 8.47 and 27.77 ± 7.67 respectively. More details were in Table 1.

**Table 1:** Sociodemographic information of participants

Sociodemographic Characters	Overall ***N*** = 3063	Gender		***p***
		Male (*n* = 1327)	Female (*n* = 1736)	
**Age (mean, SD)**	37.73 (13.38)	38.25 (13.25)	37.34 (13.48)	0.062
**Marriage**^a^				< 0.05
Singled	1049	419	630	
Married	2014	908	1106	
**Education**				0.27
High school and below	611	264	347	
College degree	1999	882	1117	
Master’s degree and above	453	181	272	
**Occupation**				0.54
Front line medical personnel	216	93	123	
General medical personnel	348	149	199	
Employed	2410	1053	1357	
Unemployed	89	32	57	
**Health status**^a^				0.12
Healthy	2416	1029	1387	
Any diseases	647	298	349	
**Distance to Hubei Province**				0.28
Hubei province	63	26	37	
First class areas	1084	446	638	
Second class areas	984	445	539	
Third class areas	932	410	522	
**Quarantine status**^a^				0.23
Quarantine for treatment	5	4	1	
Quarantine for medical observation	839	357	482	
Home quarantine	2219	966	1253	
**Number of people living together**^a^				< 0.05
Alone	225	120	105	
2–5	2615	1096	1519	
6 and above	223	111	112	
**Time spent on seeking for COVID-19 related information**				0.23
<30 min	984	447	537	
30-60 min	1445	618	827	
> 60 min	634	262	372	
**Main source of COVID-19 related information**				< 0.05
Traditional media	126	68	58	
Social media	2937	1259	1678	
**Adaption**				< 0.05
Very adapted	1746	724	1022	
Adapted	741	313	428	
Not adapted	576	290	286	
**PHQ-2** (Mean, sd)	1.30 (1.41)	1.27 (1.44)	1.33 (1.39)	0.30
Not depression	2630	1129	1501	0.28
Depression	433	198	235	
**GAD-2** (Mean, sd)	1.28 (1.44)	1.18 (1.43)	1.35 (1.44)	< 0.05
Not anxiety	2657	1165	1492	0.14
Anxiety	406	162	244	
**Resilience** (Mean, sd)	28.58 (8.08)	29.63 (8.47)	27.77 (7.67)	< 0.05
**Stress** (Mean, sd)	3.45 (2.39)	3.34 (2.37)	3.53 (2.39)	< 0.05

Note: ^a^ indicates the Fisher’s Exact Test

### Linear regression analysis of depression

As showed in Table 2, after univariate linear regression analysis, we found participants’ age, marriage status, occupation, adaption, resilience and stress were associated with depression. Then, we included these variables into multivariate linear regression analysis (Model 1).

**Table 2:** Results of univariate linear regression analysis for PHQ-2 and GAD-2

Sociodemographic Characters	PHQ-2 score			GAD-2 score		
	Standardized beta coefficient	95%CI		Standardized beta coefficient	95%CI	
		Lower	Upper		Lower	Upper
**Age (mean, SD)**	− 0.12	− 0.017	− 0.0091	0.0056	− 0.0032	0.0044
**Gender**						
Male	–	–	–	–	–	–
Female	0.019	− 0.048	0.15	0.058	0.065	0.27
**Marriage**						
Singled	--	–	–	–	–	–
Married	−0.079	− 0.34	− 0.13	0.010	− 0.076	0.14
**Education**						
High school and below	–	–	–	–	–	–
College degree	− 0.027	− 0.21	0.048	− 0.092	− 0.41	− 0.15
Master’s degree and above	− 0.017	− 0.24	0.11	− 0.078	−0.49	− 0.14
**Occupation**						
Front line medical personnel	–	–	–	–	–	–
General medical personnel	0.0048	− 0.22	0.26	− 0.0069	− 0.28	0.21
Employed	−0.021	− 0.12	0.27	0.025	−0.11	0.29
Unemployed	0.051	0.083	0.78	0.038	−0.030	0.68
**Health status**						
Healthy	–	–	–	–	–	–
Any diseases	0.023	− 0.042	0.20	0.045	0.034	0.28
**Distance to Hubei Province**						
Hubei province	–	–	–	–	–	–
First class areas	0.038	− 0.25	0.47	−0.012	− 0.40	0.33
Second class areas	0.015	−0.32	0.40	−0.031	−0.46	0.27
Third class areas	0.012	−0.32	0.40	−0.046	−0.51	0.22
**Quarantine status**^a^						
Quarantine for treatment	–	–	–	–	–	–
Quarantine for medical observation	−0.047	−1.39	1.09	0.070	−1.04	1.49
Home quarantine	−0.11	−1.59	0.88	0.0073	−1.23	1.28
**Number of people living together**^a^						
Alone	–	–	–	–	–	–
2–5	−0.029	−0.31	0.076	−0.016	− 0.26	0.13
6 and above	−0.0068	−0.30	0.23	0.014	−0.19	0.34
**Time spent on COVID-19 related information**						
<30 min	–	–	–	–	–	–
30-60 min	−0.032	− 0.21	0.023	0.072	−0.095	0.14
> 60 min	−0.0089	− 0.17	0.11	0.10	0.21	0.50
**Main source of COVID-19 related information**						
Social media	–	–	–	–	–	–
Traditional media	0.0072	−0.20	0.30	−0.018	−0.39	0.12
**Adaption**						
Very adapted	–	–	–	–	–	–
Adapted	0.14	0.35	0.58	0.099	0.21	0.45
Not adapted	0.30	0.96	1.21	0.28	0.90	1.16
**Resilience** (Mean, sd)	−0.29	−0.056	−0.044	−0.27	− 0.053	−0.041
**Stress** (Mean, sd)	0.30	0.16	0.20	0.30	0.16	0.20

Note: ^a^ indicates the Fisher’s Exact Test

We found Model 1 was a significant regression equation with an adjusted R-squared of 0.20 and a F-value of 83.57 (*p* < 0.05). However, in this model, participant’s marriage status was not significantly associated with the PHQ-2 score, thus, we removed it from analysis (Model 2). Comparing with Model 1, Model 2 improved in model fitness an adjusted R-squared of 0.20 and a F-value of 93.69 (*p* < 0.05); and all variables were significantly associated with PHQ-2 score.

In this study, participants’ PHQ-2 score would decrease 0.011 point for each unit of age increased. Comparing with front line medical personnel, unemployed participants’ PHQ-2 score would be 0.044 point higher. Comparing with participants who felt very adapted, PHQ-2 score would be 0.078 and 0.24 point higher among participants who felt adapted and not adapted respectively. Meanwhile, participants’ PHQ-2 score would decrease 0.19 point for each unit of resilience increased, and it would increase 0.21 point for each unite of stress increased. More details were showed in Table 3.

**Table 3:** Results of multivariate linear regression for PHQ-2 score

Sociodemographic Characters	Model 1			Model 2		
	Standardized beta coefficient	95%CI		Standardized beta coefficient	95%CI	
		Lower	Upper		Lower	Upper
**Age (mean, SD)**	−0.056	−0.0097	− 0.00099	−0.076	− 0.011	−0.0047
**Marriage**						
Singled	–	–	–	–	–	–
Married	− 0.032	−0.22	0.028	–	–	–
**Occupation**						
Front line medical personnel	–	–	–	–	–	–
General medical personnel	0.0090	−0.26	0.18	−0.0079	− 0.25	0.18
Employed	0.0062	−0.16	0.20	0.0071	−0.15	0.20
Unemployed	0.044	0.055	0.68	0.044	0.060	0.69
**Adaption**						
Very adapted	–	–	–	–	–	–
Adapted	0.077	0.14	0.36	0.078	0.15	0.37
Not adapted	0.25	0.77	1.00	0.24	0.76	1.00
**Resilience**	−0.19	−0.038	−0.027	−0.19	−0.038	− 0.027
**Stress**	0.22	0.11	0.15	0.21	0.11	0.15
**Adjusted R-Squared**	0.20			0.20		
**F**	83.57			93.69		
***p***	< 0.05			< 0.05		

### Linear regression analysis of anxiety

As showed in Table 2, after univariate linear regression analysis, we found participants’ marriage status, education, health status, time on COVID-19 related information, adaption, resilience and stress were associated with anxiety. Then, we included these variables into multivariate linear regression analysis (Model 3).

We found Model 3 was a significant regression equation with an adjusted R-squared of 0.20 and a F-value of 71.75 (*p* < 0.05). However, in this model, participant’s health status was not significantly associated with the PHQ-2 score, thus, we removed it from analysis (Model 4). Comparing with Model 3, Model 4 improved in model fitness an adjusted R-squared of 0.20 and a F-value of 79.46 (p < 0.05); and all variables were significantly associated with GAD-2 score.

In this study, the GAD-2 score would be 0.042 point higher in females than in males. Comparing with participants who only received high school education or less, the GAD-2 score would be 0.078 and 0.073 point lower among participants who received college degree and who received masters’ degree and above. Comparing with participants spending less than 30 min on COVID-19 related information, the GAD-2 score would be 0.10 point higher among who would spend more than 60 min on such information. Comparing with participants who felt very adapted, the GAD-2 score would be 0.046 and 0.22 point higher among participants who felt adapted and not adapted respectively. Meanwhile, participants’ GAD-2 score would decrease 0.17 point for each unit of resilience increased, and it would increase 0.23 point for each unite of stress increased. More details were showed in Table 4.

**Table 4:** Results of multivariate linear regression for GAD-2 score

Sociodemographic Characters	Model 3			Model 4		
	Standardized beta coefficient	95%CI		Standardized beta coefficient	95%CI	
		Lower	Upper		Lower	Upper
**Gender**						
Male	–	–	–	–	–	–
Female	0.043	0.029	0.22	0.042	0.027	0.21
**Education**						
High school and below	–	–	–	–	–	–
College degree	−0.078	−0.35	−0.12	−0.078	−0.36	−0.12
Master’s degree and above	−0.070	−0.44	− 0.12	−0.073	− 0.45	−0.13
**Health status**						
Healthy	–	–	–	–	–	–
Any diseases	0.024	−0.029	0.20	–	–	–
**Time spent on seeking for COVID-19 related information**						
<30 min	–	–	–	–	–	–
30-60 min	−0.0028	− 0.11	0.10	− 0.0017	−0.11	0.10
> 60 min	0.10	0.23	0.49	0.10	0.24	0.50
**Adaption**						
Very adapted	–	–	–	–	–	–
Adapted	0.047	0.043	0.27	0.046	0.042	0.27
Not adapted	0.22	0.70	0.95	0.22	0.69	0.94
**Resilience**	−0.17	−0.036	−0.024	− 0.17	−0.036	− 0.024
**Stress**	0.23	0.12	0.16	0.23	0.12	0.16
**Adjusted R-Squared**	0.19			0.19		
**F**	71.75			79.46		
**p**	< 0.05			< 0.05		

## Discussion

In this study, we reported the prevalence of depression was 14.14% and the prevalence of anxiety was 13.25%. Gender differences were observed in the severity of anxiety symptoms, self-rate stress and the ability of resilience to stress: females were experiencing more severe stress and anxiety symptoms, while males showed better resilience to stress. And our analysis showed that being older and greater resilience to stress would decrease the severity of depression, while being unemployed, less adapted to the epidemic and experiencing higher stress would increase the severity. We also showed that receiving higher education and greater resilience to stress would decrease the severity of anxiety, while being female, spending more than 60 min on COVID-19 related information, less adapted to the epidemic and experiencing higher stress would increase the severity.

The prevalence of depression and anxiety in this study was higher than the lasted results from the China Mental Health Survey reported in 2019, in which the prevalence of depression and anxiety was 6.8 and 7.6% respectively [12]. The results are consistent with previous studies reporting increased mental health problems after major public health emergent events including the SARS outbreak and the Wenchuan and Lushan earthquake [44–48]; meanwhile, we noticed that the prevalence of depression and anxiety in this study was lower than that in these studies. The differences may be resulted from the following explanations. First, provincial governments activated the Leve-1 Public Health Emergency Responses (the highest level of public health emergency response in China), which included mental health crisis interventions, for example, counseling through hotlines and social media (especially on TikTok, Weibo and WeChat), posters in communities, mental health promotion programs on TV, radio and social media. Second, we believed the early awareness of the outbreak prepared the public, given the fast spread of limited information related to COVID-19 in Wuhan through social media in early January when officials claimed there wasn’t clear evidence supporting it was human-to-human transmission. Third, this study was conducted at the end of February when new COVID-19 cases dropped indicating the rapid spread of infections has been contained, which relieved psychological stress and decreased depression and anxiety symptoms in return.

Social media was the main source of updating the COVID-19 related information for participants in this study, we observed the gender difference that the rate of using traditional media as main source of information in males. However, there was no gender differences in time spent on seeking for COVID-19 related information; while we found when participants spent more than 60 min on seeking the COVID-19 related information, they would significantly show more severe anxiety symptoms than those spent less than 60 min consistent with previous meta-analysis [49]. However, we did not investigate the relationship between the Coronavirus ‘infodemic’ (related to the spread of false report, rumors and disinformation), emphasized by the WHO [50], and mental health.

In this study, it was worth noting that the distance between participants’ residence area and Hubei Province did not associate with depression and anxiety. First, the immediate implementation of the Leve-1 Public Health Emergency Responses in all provinces and the daily updates about surveillance, active cases, cured cases and even deaths have helped the public to clarify infodemic and avoid panic. Second, there were increasing number of psychologists and psychiatrists sharing strategies against stress during the epidemic by social media, especially TikTok, Weibo and live stream, which were not restricted to regions. For example, psychologists from Shenzhen Kangning Hospital/Shenzhen Mental Health Center have hosted series of programs (podcasts, videos) for front line medical staff, residents, parents, elders and children dealing with psychological stress [51]. Third, strategies fighting against the epidemic did not differ among provinces that have resulted in similar impact on life and work.

The National Health Commission of China has released a guideline in late January 2020, and requires all local authorities to provide four types of mental health intervention targeting on patients and frontline staff of COVID-19, close contacts under quarantine, family members of the diagnosed, and the general population, respectively [52]. In this guideline, social media or the Internet are the vehicle for public propaganda to deliver psychological interventions and key information. However, there is no code of conducts or regulations for social media during epidemics. Based on our findings, we believe it is important to establish the code of conducts for social media to report epidemic information and implement mental health interventions; and it is equally important for internet-based social media platforms to identify users who are vulnerable to psychological distress, deliver mental health care for whom in need, and improve information streaming mechanism to protect users from excessive exposure to epidemic information.

There are some limitations about the current study. First, this is a cross-sectional study for rapid assessment for depression and anxiety during the epidemic, and we are unable to investigate causal relationships. Second, the convenient sampling method and on-line recruitment could lead to highly vulnerable to selection bias and high level of sampling error. We would have sampled participants from a particular social network. The first wave participants were more likely to be health professionals (medical and academic staff and students), and the rest participants were in their social network, thus, the sample was limited to represent the general population of social media users. Meanwhile, we did not record how many social media users we have approached, how many of them decided not to participate. Nevertheless, the results showed the demographic distribution of our study sample resembled the general population of social media users in China. Third, we conducted the survey 1 month after the outbreak that we could not investigate the acute mental health impact of the epidemic.

## Conclusion

The findings show the increased prevalence of depression and anxiety in Chinese population during the COVID-19 epidemic, and females are experiencing more severe anxiety symptoms than men. As social media is current the main resource of information related to COVID-19, interventions should be implemented to help users to limit the time they spend on social media and to obtain key information related to the epidemic from authoritative and authentic resource, to avoid infodemic and prevent mental health problems.

## Data Availability

The data on which this manuscript is based are not available to public. The data from this study are under certain restrictions according to the Military Logistics Research Foundation of China and always under the supervision of the principal investigator of the study. Thus, there are access restrictions to the data. However, at any time, researchers can contact the principal investigator (Kangxing Song, skx301@126.com) for data sharing.

## Abbreviations

COVID-19: 2019 Novel Coronavirus Disease
WHO: World Health Organization
IPV: Intimate partner violence
PHQ-2: The 2-item Patient Health Questionnaire
PHQ-9: The 9-item Patient Health Questionnaire
GAD-2: The 2-item Generalized Anxiety Disorder Scale
GAD-7: The 7-item Generalized Anxiety Disorder Scale
CD-RISC-10: The Chinese version of the 10-item Connor-Davidson Resilience Scale
ANOVA: Analysis of variance
